# New plastome from an introduced *Salvia yunnanensis* C.H.Wright (Lamiaceae) challenges previous phylogenetic interpretations

**DOI:** 10.1080/23802359.2025.2576515

**Published:** 2025-10-22

**Authors:** Junfei Liu, Weihan Yuan, Liqiang Wang, Hai Zhang

**Affiliations:** ^a^ College of Pharmacy, Heze University, Mudan District Heze City, Shandong Province, P.R. China; ^b^ Department of Research and Development, Shandong Kunhetang Pharmaceutical Co. Ltd, Cao County Heze City, Shandong Province, P.R. China; ^c^ Quality Control Department, Shandong Huaxin Pharmaceutical Group Co. Ltd, Mudan District Heze City, Shandong, P.R. China

**Keywords:** Lamiaceae, next-generation sequencing technology, phylogenetic analysis, *Salvia yunnanensis*, third-generation sequencing technology

## Abstract

*Salvia yunnanensis* C.H. Wright 1896, a species in the Lamiaceae family, is a medicinal plant whose roots are used as herbal medicine under the name "Zidanshen." To provide useful genetic information on this plant, in this study, we assembled another complete plastome of a successfully introduced *S. yunnanensis* using a combination of next-generation sequencing (NGS) and third-generation sequencing (TGS) data. The plastome is 151,486 bp and comprises a large single-copy (LSC) region of 82,759 bp, a small single-copy (SSC) region of 17,577 bp, and a pair of inverted repeat (IR) regions of 25,575 bp each. It encodes 132 genes, which include 87 protein-coding genes, eight rRNA genes, and 37 tRNA genes. The guanine-cytosine (GC) content of the genome is 38.01%. Phylogenetic analysis revealed that *S. yunnanensis* was closely related to *S. honania*. Genome assembly benefits from the strengths of NGS and TGS data, resulting in high-quality assembly. It showed 99.53% similarity to the previously reported plastome (MN341012), with a slight increase in length. However, the gene count and GC content remained unchanged. Phylogenetic analysis of whole plastomes revealed that *S. yunnanensis* in this study formed a monophyletic clade distinct from *Salvia honania* but did not group with any publicly available *S. yunnanensis* plants. This newly sequenced plastome provides a valuable resource for the introduction and domestication of this plant resource in the future.

## Introduction

**Figure 1.**
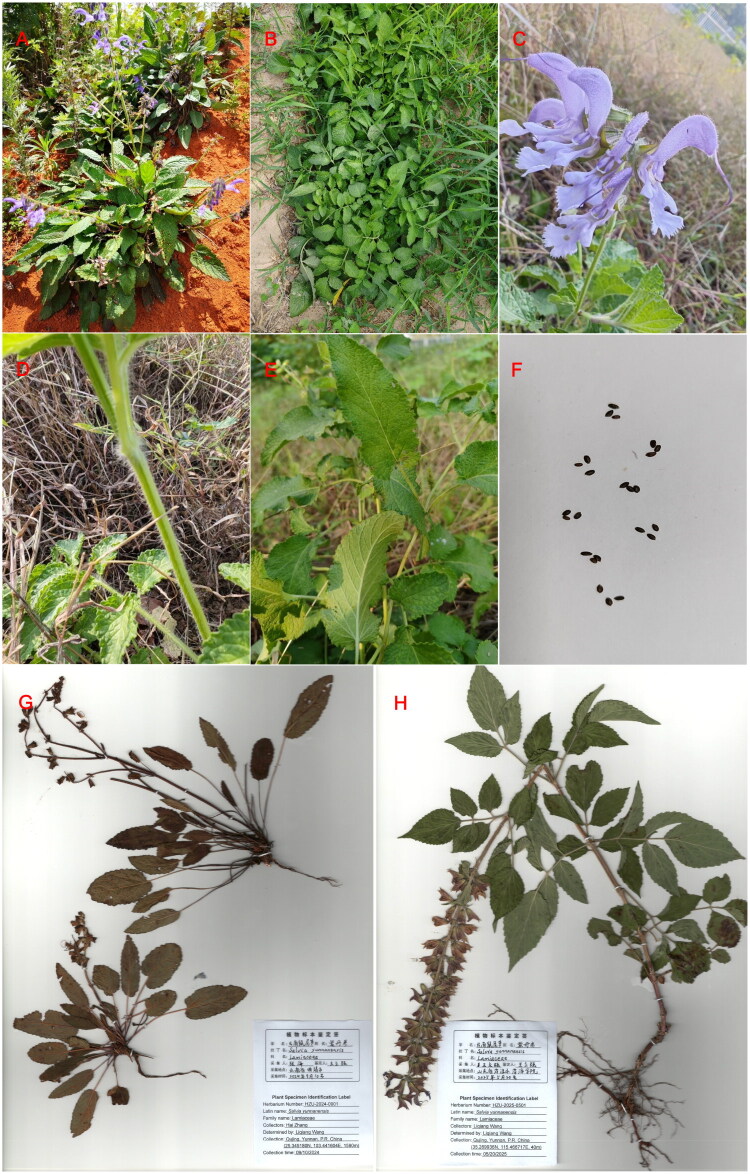
The Morphology of *Salvia yunnanensis*. (A) Habitat photos of *S. yunnanensis* from its native location in Qujing, Yunnan, China (25.345180° N, 103.441604° E, 1590 m). The key features include petioles, stems and flowers covered with fine hairs and long-oval leaves, either simple or compound. Photo credit: Hai Zhang. (B) *Salvia yunnanensis* cultivated in Heze, Shandong, China, after domestication. (C) Flowers after domestication, with petals densely covered in fine hairs. The verticillasters consisted of 4–6 flowers spaced apart, forming terminal racemes or racemose panicles that were 7–13 cm long. The corolla is bluish-purple, 2.5–3 cm long, and pubescent on the exterior. (D) Stems post-domestication, densely covered in fine hairs. (E) Long-oval leaves post-domestication. (F) Seeds produced after domestication. (G) A specimen of *S. yunnanensis* from its native habitat in Qujing, Yunnan. (H) A specimen of *S. yunnanensis* was introduced and cultivated in the Heze region. Photos B-F were captured by Liqiang Wang at 35.269936° N, 115.466717° E, 40 m. Photography, species identification, seed collection, and specimen production were all completed by Liqiang Wang.

*Salvia yunnanensis* C.H. Wright 1896, a member of the Lamiaceae family, is a traditional medicinal plant widely used in southwestern China. Its roots, known as Zidanshen, are used in herbal medicine (Ma et al. 2021). The plant is locally called “Dainaise” by the Yi ethnic group (Guan et al. 2020) and is found primarily in eastern, central, and western Yunnan Province. It grows on grassy slopes, forest edges, roadsides, and dry woodlands at elevations of 1,800–2,900 m. Native habitats feature red soil (Figure 1(A)), with average annual temperatures of 14.8–21.5 °C, extreme temperatures ranging from −13 °C to 36 °C, annual rainfall of 614.5–1,406.8 mm, a frost-free period of 210–330 days, and annual sunshine of 1,200–2,400 h.

Zidanshen has a high demand for pharmaceutical production, but overharvesting has drastically reduced the populations of wild *S. yunnanensis*, driving the species toward extinction (Chen et al. 2018). To address this, we transplanted *S. yunnanensis* from Yunnan to Heze City, Shandong Province. In Heze, the plant grows in yellow soil (Figure 1(B)) and shows slight morphological changes (Figures 1(C–E)) but retains key characteristics such as oblong leaves and dense white hairs on stems and flowers. The domesticated plants also produce seeds (Figure 1(F)).

Zidanshen shares medicinal properties with Danshen (*Salvia miltiorrhiz*a), containing phenolic acids (e.g. tanshinones, caffeic acid, rosmarinic acid, and salvianolic acid B) and diterpenoids with antioxidant, anti-inflammatory, antithrombotic, and cardiovascular benefits (Ma et al. 2021). *Salvia yunnanensis* has twice the phenolic acid content of *S. miltiorrhiza*, with salvianolic acid B being the most abundant (Qian et al. 2002; Zhang et al. 2010). It also produces unique diterpenoids such as Zidanshenone, which exhibit antiplatelet, anti-HIV, and myocardial ischemia protection properties, outperforming Danshen in inhibiting the aggregation of platelets (Ma et al. 2021). The medicinal value of *S. yunnanensis* has encouraged research on its chemical composition, DNA barcoding (Wang et al. 2007; Zhang et al. 2012), and plastome.

By 2022, five plastomes of *S. yunnanensis* were available in GenBank, including one (MN341012) fully assembled only using next-generation sequencing (NGS) data (Tao et al. 2019). However, two important knowledge gaps hinder genetic research. (1) Technical limitations: Existing plastome assemblies relying solely on NGS data face challenges in resolving repetitive regions and GC-biased areas (Bolger et al. 2014). (2) Effect of introduction effects: The introduction process may alter the selective pressures on genomic variation, leading to deviations in plastome divergence patterns between *S. yunnanensis* and its close relatives (particularly *Salvia honania*). This highlights the need to account for introduction-induced genetic variation when delimiting species boundaries within the *Salvia* subgenus *Sclarea*.

To make genetic research on *S. yunnanensis* during introduction more accurate, in this study, we newly assembled the plastome *via* both NGS and third-generation sequencing (TGS) data and reanalyzed its structure and phylogenetic relationships.

## Materials and methods

Samples of *S. yunnanensis* (Figure 1(B)) were collected from the campus of Heze University, Heze City, Shandong Province, China (35.269936° N, 115.466717° E, 40 m). A voucher specimen was deposited in the Herbarium of Heze University under specimen number HZU-2025-0501 (Figures 1(G–H)). All tasks pertaining to sample collection, including photography, species identification, seed gathering, and specimen preparation, were conducted by Liqiang Wang (contact: lys832000@163.com). The characteristic feature of *S. yunnanensis* is that the petioles, stems, and flowers are covered with fine hairs. During the seedling stage, the leaves are simple and then rapidly develop into compound leaves. The leaf has an elongated and elliptical shape. The verticillasters consist of 4–6 flowers spaced apart, forming terminal racemes or racemose panicles that are 7–13 cm long. The corolla is bluish-purple, 2.5–3 cm long, and pubescent on the exterior.

Tender leaves from healthy plants were used to extract and purify whole-genome DNA for NGS and TGS using the Plant Genomic DNA Kit (Tiangen Biotech, Beijing, China). The quality of the DNA obtained was assessed, and the samples were fragmented into ∼300 bp inserts for NGS. Library preparation and sequencing were performed on the Illumina NovaSeq 6000 platform by Wuhan Benagen Technology Co.. TGS data were generated using the PromethION platform (Oxford Nanopore Technologies, Wuhan Benagen Technology Co.).

Low-quality reads from NGS and TGS were filtered using Trimmomatic (v0.39) (Bolger et al. 2014) and NanoPack2 (De Coster and Rademakers 2023), respectively. The first plastome was assembled using GetOrganelle (v1.7.1) (Jin et al. 2020) with NGS data alone. The second plastome was assembled using both NGS and TGS data with Unicycler (Wick et al. 2017). The reads were filtered using BLASTn with the *S. yunnanensis* plastome (MT093187) as a reference, and the filtered reads were input into Unicycler for the second assembly. Both plastomes were annotated using CPGAVAS2 (Shi et al. 2019) and manually refined using Apollo (Pontius 2018). The annotated plastomes were submitted to GenBank *via* BankIt, and a circular genome map was generated using CPGview (Liu et al. 2023), which also produced the genome map and the structure of the cis-/trans-splicing genes. The sequencing depth of the assembled genome was calculated using the online tool DrawSeqDepth (https://github.com/wlqg1983/DrawSeqDepth).

For phylogenetic analysis, 75 common gene sequences from 35 *Salvia* species (subgenus *Sclarea*) and two outgroups (*Glechoma longituba* and *Mentha canadensis*) (Li and Mo 2019; Li et al. 2019) were aligned using MAFFT (Katoh and Standley 2016) with default settings. A maximum likelihood (ML) tree was constructed using IQ-TREE (v2.0) (Nguyen et al. 2015) with 1,000 bootstrap replicates and the TVM+F + I + R6 substitution model. The nucleotide substitution model was selected by ModelFinder (Kalyaanamoorthy et al. 2017) embedded in the IQ-TREE software.

## Results

**Figure 2.**
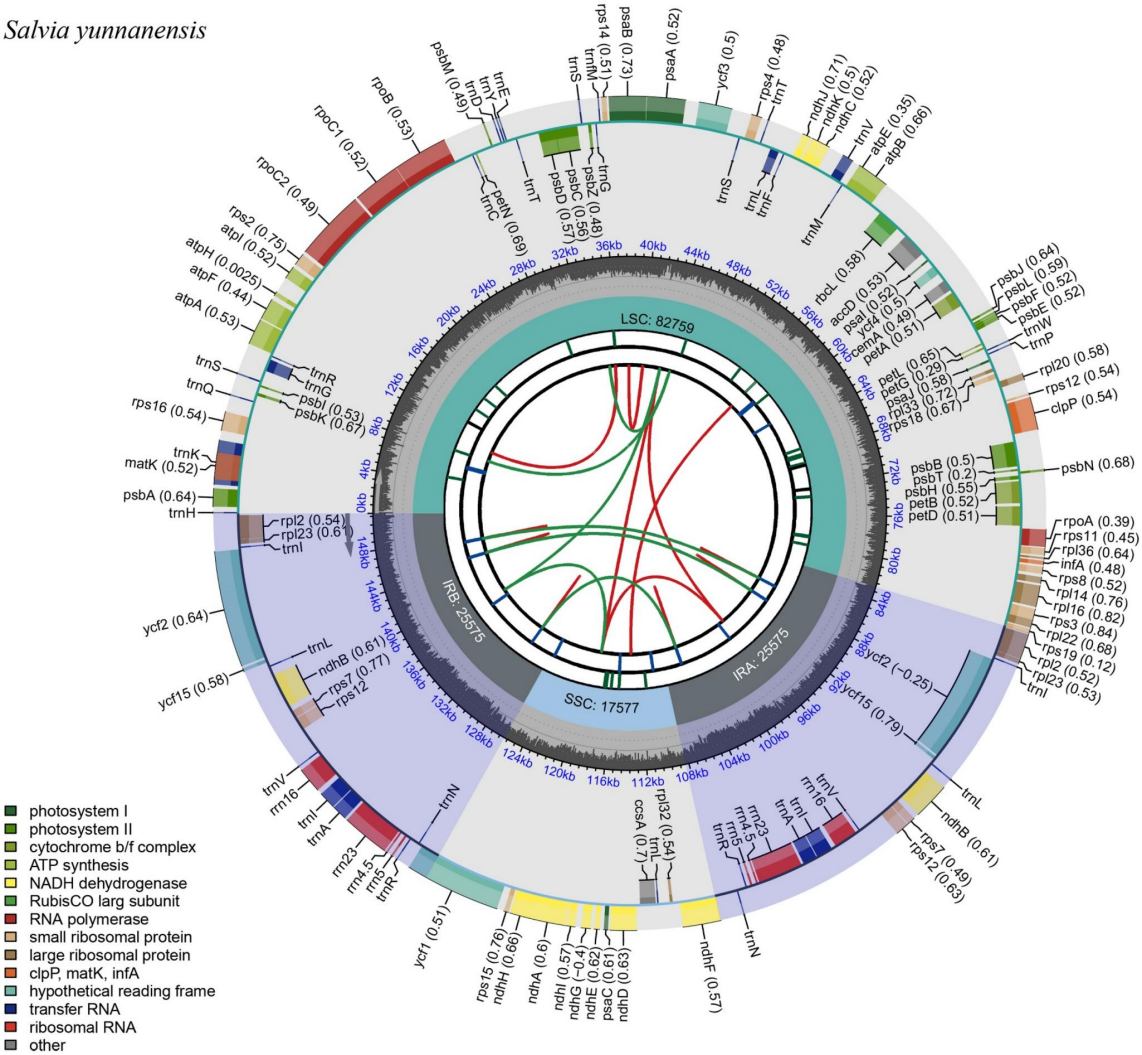
Plastome mapping of *Salvia yunnanensis* genomes assembled *via* NGS and TGS data. The species name was shown in the top left corner. The map included six tracks. From the center outward, the first track showed dispersed repeats (direct and palindromic), marked by red and green arcs. The second track displayed long tandem repeats, represented as blue bars. The third track showed short tandem repeats or microsatellites, represented as short colored bars. The fourth track included the SSC, IRa, IRb, and LSC regions. The fifth track showed the GC content across the genome. The sixth track presented genes, color-coded by function (see bottom left corner). For protein-coding genes, letters after names indicated subunits or family members; numbers denoted variants. For rRNA genes, the numbers represented the rRNA size in Svedberg units. For tRNA genes, letters indicate recognized amino acids. For unknown-function genes, the numbers referred to hypothetical coding genes. Inner genes were transcribed clockwise; outer genes are transcribed anticlockwise.

We assembled the plastomes of *S. yunnanensis* using both NGS and TGS data, as well as NGS data alone. The genomes were deposited in GenBank under accession numbers PQ524041.2 and PQ524042.1. Both plastomes, measuring 151,486 bp (Figure 2) and 151,483 bp (Figure S1), showed the typical tetrameric structure. Subsequent analysis investigated the genome assembled from combined NGS and TGS data. This plastome comprises a large single-copy region (LSC, 82,759 bp), a small single-copy region (SSC, 17,577 bp), and two inverted repeat regions (IRa and IRb, 25,575 bp each). The minimum coverage depths were 139× (Figure S2A) and 633× (Figure S2B) for NGS and TGS, respectively. In this study, the reassembled plastome showed 99.53% similarity to the previously reported genome (MN341012) based on the BLASTN comparison. The difference in length occurred mainly in the LSC region, with an increase of 138 bp, followed by the SSC region (13 bp longer) (Figure S3). Variability analysis revealed polymorphisms in intergenic regions and some protein-coding genes, such as *ycf*1, *ycf*2, *rps*19, and *rpo*A (Figure S4). Compared to MN341012.1, the genome has a 61-bp insertion mutation in the intergenic region *trn*F-GAA/*ndh*J (Table S1). This locus may serve as a molecular marker for identifying introduced *S. yunnanensis* at the species level.

**Figure 3.**
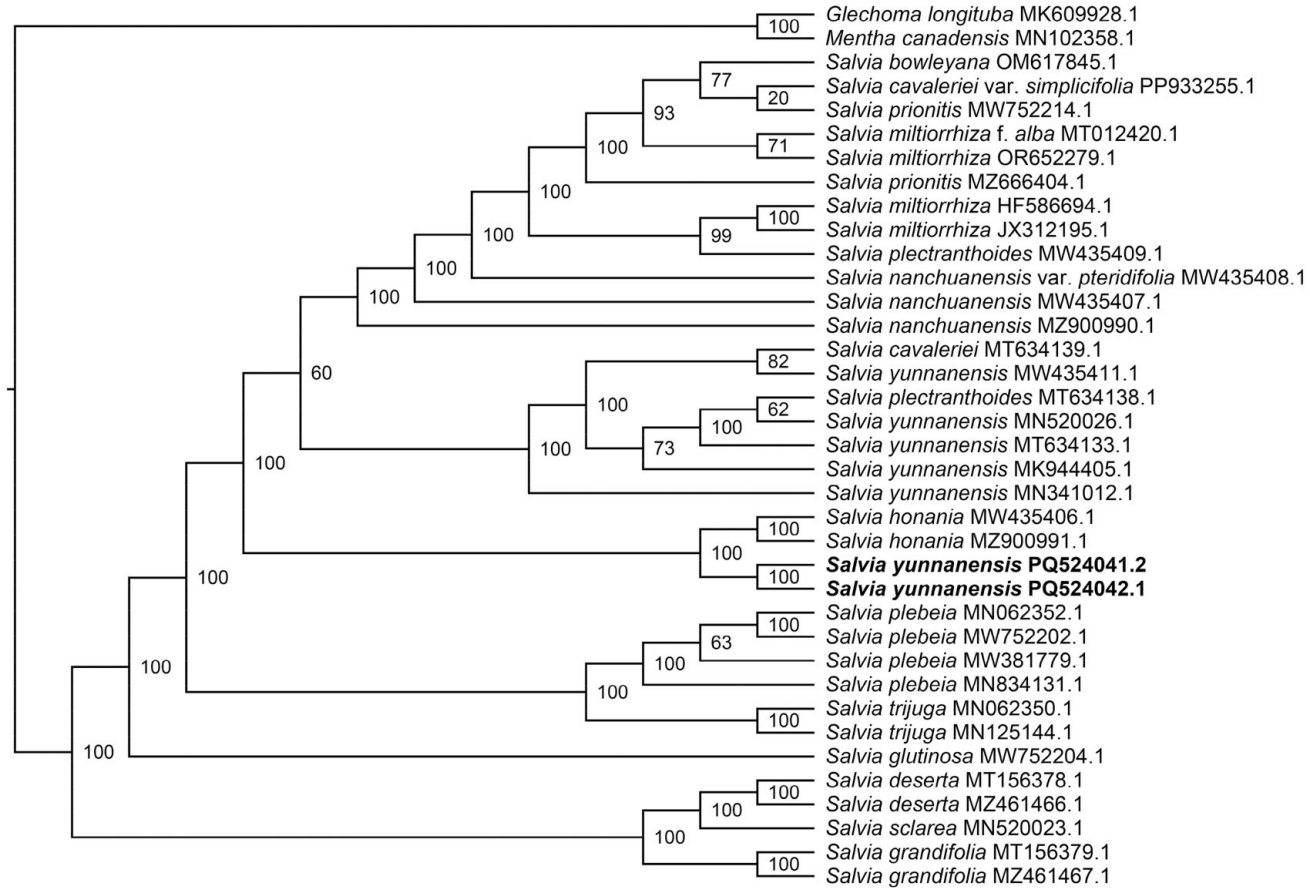
The ML phylogenetic cladogram tree of *Salvia yunnanensis* and related taxa based on common gene sequences. The bootstrap values based on 1000 replicates were shown on each node. *Salvia* plants (*n*** **=** **35) and two outgroups were selected for reconstructing the ML tree. New *S. yunnanensis* plastomes (PQ524041, assembled from NGS and TGS data; PQ524042, assembled from NGS data) in this study are labeled in bold font. The two outgroups were *glechoma longituba* (MK609928.1) (Li and Mo 2019) and *mentha canadensis* (MN102358.1) (Li et al. 2019). The other 33 *Salvia* plastomes were from *S. bowleyana* (OM617845.1), *S. cavaleriei* var. *simplicifolia* (PP933255.1), *S. cavaleriei* (MT634139.1) (Wu et al. 2021), *S. prionitis* (MW752214.1, MZ666404.1) (Su et al. 2022), *S. miltiorrhiza* f. *alba* (MT012420.1), *S. miltiorrhiza* (HF586694, JX312195, OR652279) (Qian et al. 2013), *S. plectranthoides* (MW435409.1, MT634138.1) (Su et al. 2022), *S. nanchuanensis* (MW435407.1, MZ900990.1), *S. nanchuanensis* var. *pteridifolia* (MW435408), *S. yunnanensis* (MK944405, MN341012, MN520026, MT634133, MW435411) (Tao et al. 2019), *S. honania* (MW435406.1, MZ900991.1) (Wang et al. 2022), *S. plebeia* (MN062352.1, MW381779.1, MW752202.1, MN834131.1) (Cui et al. 2020), *S. trijuga* (MN062350.1, MN125144.1) (Du et al. 2021), *S. glutinosa* (MW752204.1) (Huang et al. 2024), *S. deserta* (MT156378.1, MZ461466.1) (Li et al. 2013), *S. sclarea* (MN520023.1) (Zhao et al. 2020), *S. grandifolia* (MT156379.1, MZ461467.1) (Huang et al. 2024).

The plastome contains 132 genes (114 unique), including 87 protein-coding genes (80 unique), eight rRNA genes (four unique), and 37 tRNA genes (30 unique), with a GC content of 38.01%. Among these genes, 23 have one or two introns and are cis-splicing. Genes such as *rps*16, *atp*F, *rpo*C1, *pet*B, *pet*D, *rpl*16, *rpl*2 (×2), *ndh*B (×2), *ndh*A, *trn*K-UUU, *trn*G-UCC, *trn*L-UAA, *trn*V-UAC, *trn*I-GAU (×2), and *trn*A-UGC (×2) contain one intron (Figure S3). The genes *rps*12 (×2), *clp*P, and *ycf*3 have two introns (Figure S5). The *rps*12 gene is trans-spliced, with its downstream 3′ end located in the IR region and contains an intron at each 3′ end (Figure S6). The *ycf*1 gene has a pseudogene copy because it crosses the boundary between SSC and IR. To analyze the phylogenetic relationships of *S. yunnanensis*, plastome sequences from 34 *Salvia* species and two outgroups were used to construct an ML tree. In the ML tree, the two *S. yunnanensis* plants (PQ524041 and PQ524042) formed a monophyletic branch, closely related to *S. honania* with 100% bootstrap support (Figures 3 and S7).

## Discussion and conclusion

In this study, the plastome of *S. yunnanensis* (introduced from Yunnan to Shandong cultivation) was reassembled and characterized using NGS and TGS data, as well as NGS data alone. The two plastomes differ by only three indels. Although the plastome (PQ524041) is 148 bp longer than the previously reported *S. yunnanensis* plastome (MN341012) (Tao et al. 2019), the two plastomes are highly similar (99.53%), with identical gene counts and GC contents. The reassembled plastome combines the benefits of long-read TGS data and the high accuracy of NGS data, resulting in a more reliable assembly, supported by coverage depth.

The genus *Salvia* is divided into four morphologically defined subgenera (the subgenus *Salvia, Sclarea, Calosphace*, and *Leonia*) (Tomou et al. 2024). To analyze the phylogenetic position of *S. yunnanensis* in the subgenus *Sclarea*, complete plastomes from subgenus *Sclarea* species were aligned to construct an ML tree. The *S. yunnanensis* plants in this study formed a monophyletic group with *S. honania*, which agrees with previous findings (Du et al. 2022). However, they did not form a monophyletic branch with five previously studied *S. yunnanensis* plants, which instead formed a group with *S. plectranthoides* and *S. cavaleriei*. A similar pattern was found in *S. miltiorrhiza*, *S. prionitis* and *S. nanchuanensis*, which also did not form distinct monophyletic groups. This phenomenon has been reported in other studies involving *Epimedium mikinorii* and *E. pseudowushanense* (Zhang et al. 2020).

After *S. yunnanensis* was introduced, its altered environment may have driven divergent plastome evolution compared to that of the native populations, explaining their phylogenetic separation in the analyses. Plastome variation influenced by domestication has also been observed in *Gossypium* spp. (Yan et al. 2024). Furthermore, ML phylogenetic analysis based on ITS sequences provided additional support for the finding (Figure S8). The introduced *S. yunnanensis* specimens in this study showed closer phylogenetic affinity to *S. honania* than to previously characterized *S. yunnanensis* accessions (Figure S8). The reported results provided a scientific basis for the introduction and domestication of this plant resource.

## Supplementary Material

Supplemental Material

## Data Availability

The complete plastome sequence of *S. yunnanensis* in this study was submitted to the NCBI database under the accession numbers PQ524041.2 and PQ524042.1. The associated BioProject is PRJNA1214223. The BioSample, and SRA numbers of NGS data are SAMN46363610 and SRR32111878, respectively. The BioSample and SRA numbers of the TGS data are SAMN46363653 and SRR32128869, respectively.
