# Supplementary material for: New plastome from an introduced Salvia yunnanensis C.H.Wright (Lamiaceae) challenges previous phylogenetic interpretations
Source: Mitochondrial DNA B Resour. 2025 Oct 22;10(11):1056–61. doi: 10.1080/23802359.2025.2576515 (PMC12548074; doi:10.1080/23802359.2025.2576515)
Supplement: Supplemental Material [file TMDN_A_2576515_SM5885.docx]

**Table S1.** Indels between two plastomes numbered MN341012.1 and PQ524041.2.

| Start | End | Length (bp) | Sequences | Indel |
| --- | --- | --- | --- | --- |
| 6139 | 6157 | 19 | ACTAATAGAATATATATAA | Insertion |
| 27177 | 27201 | 25 | AAAAAGATTCTTGGGAGATAAAGAA | Deletion |
| 35716 | 35738 | 23 | AAAAAATACAATTCATTAGATTC | Insertion |
| 47857 | 47917 | 61 | GCGAGATACGTATTGATTTGGAATATGGATCATAATCCATACTTTTCTTTTATCTATCTTG | Insertion |
| 58428 | 58454 | 26 | TTAGCTTATCCGAATCTTATCCGAATC | Insertion |
| 64700 | 64737 | 38 | AAAACATAGTATTCCAAAAAAATGGAATTCAAAATAAT | Insertion |
| 80672 | 80703 | 35 | AACGTCCTAATAAATCTTTATTGTCTTTTATC | Deletion |


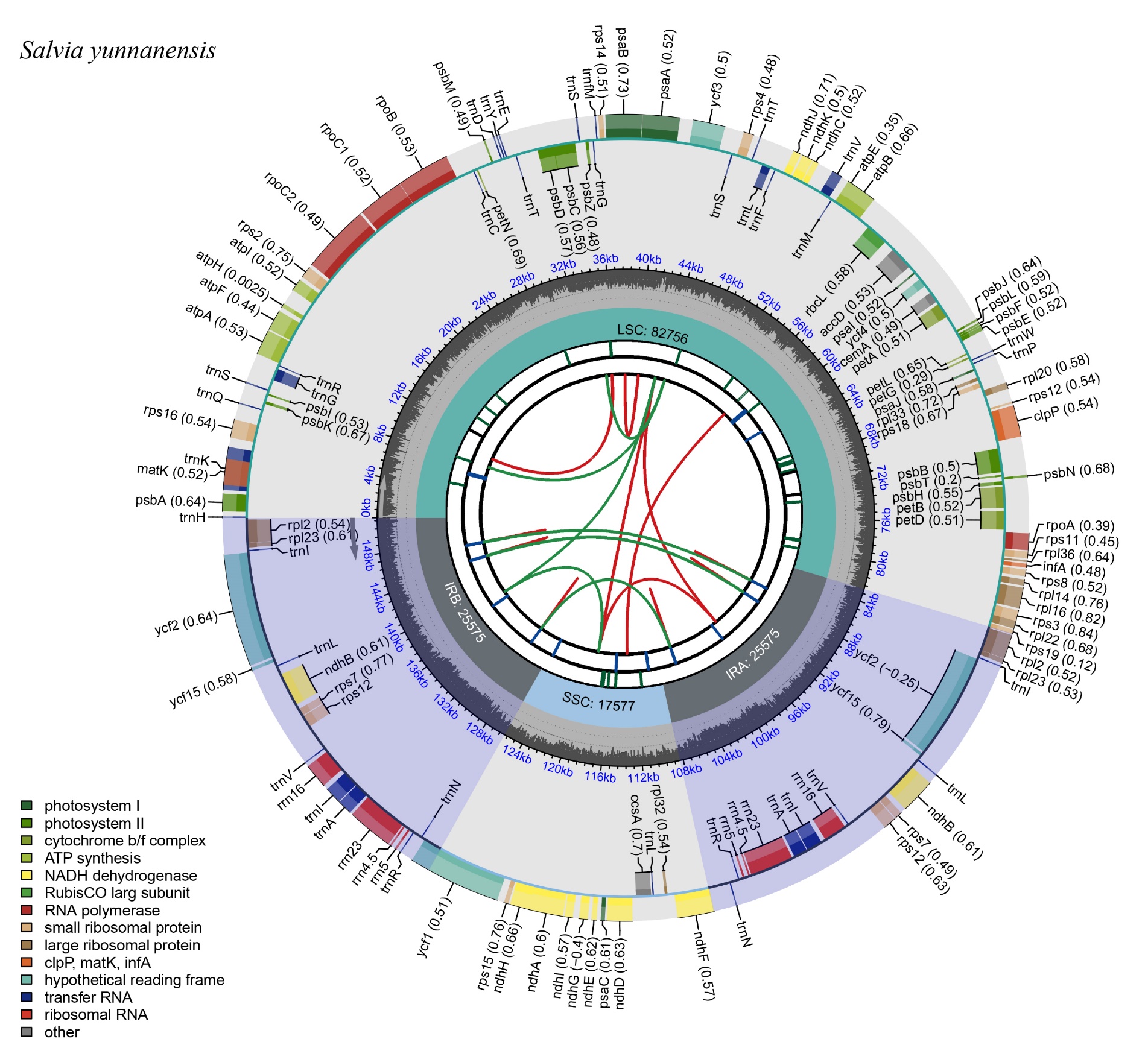


**Figure S1.** Plastome mapping of *Salvia yunnanensis* assembled via NGS data. The species name was shown in the top left corner. The map included six tracks. From the center outward, the first track showed dispersed repeats (direct and palindromic), marked by red and green arcs. The second track displayed long tandem repeats, represented as blue bars. The third track showed short tandem repeats or microsatellites, represented as short colored bars. The fourth track included the SSC, IRa, IRb, and LSC regions. The fifth track showed the GC content across the genome. The sixth track presented genes, color-coded by function (see bottom left corner). For protein-coding genes, letters after names indicated subunits or family members; numbers denoted variants. For rRNA genes, the numbers represented the rRNA size in Svedberg units. For tRNA genes, letters indicated recognized amino acids. For unknown-function genes, the numbers referred to hypothetical coding genes. Inner genes were transcribed clockwise; outer genes were transcribed anticlockwise.


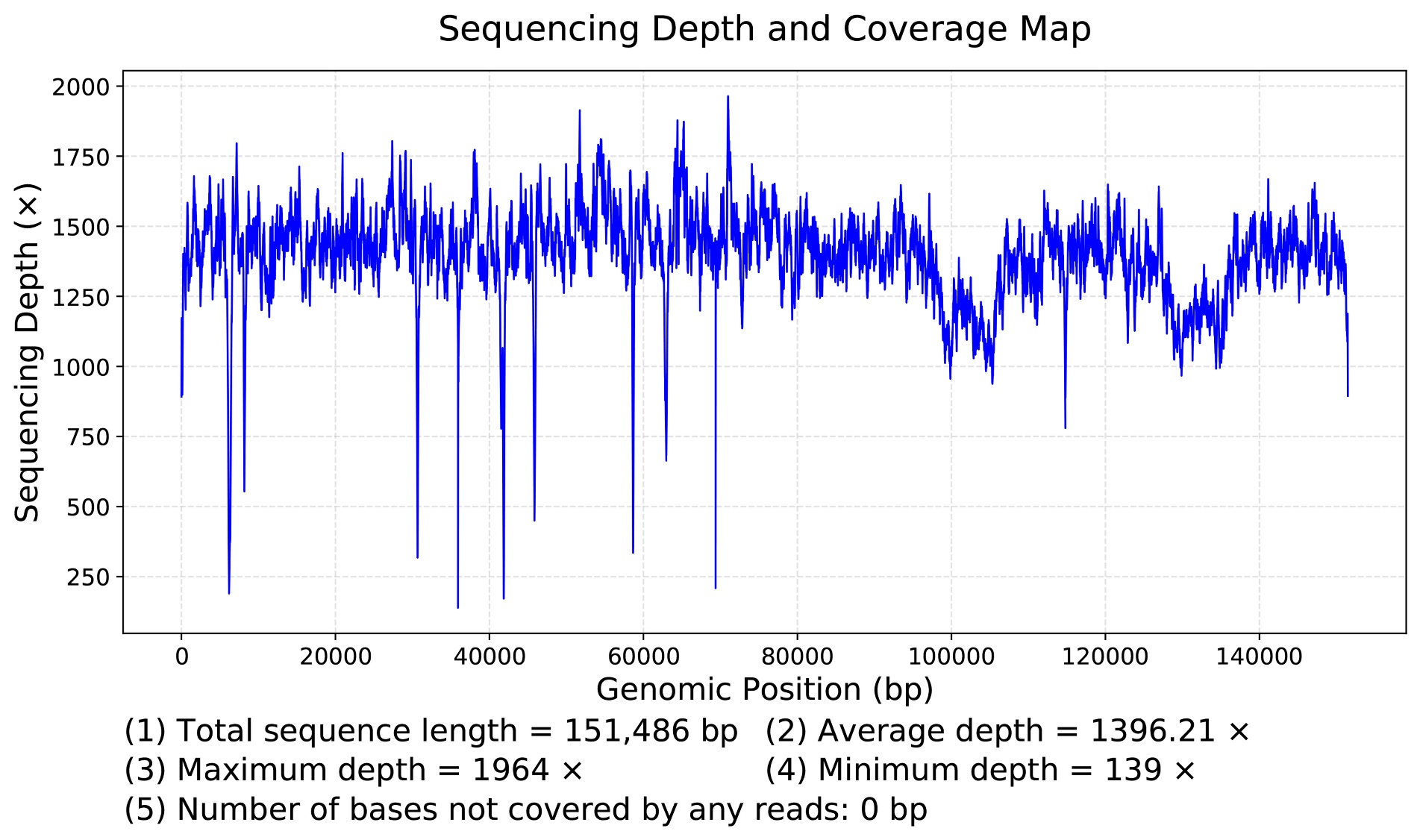


**A**

**B**


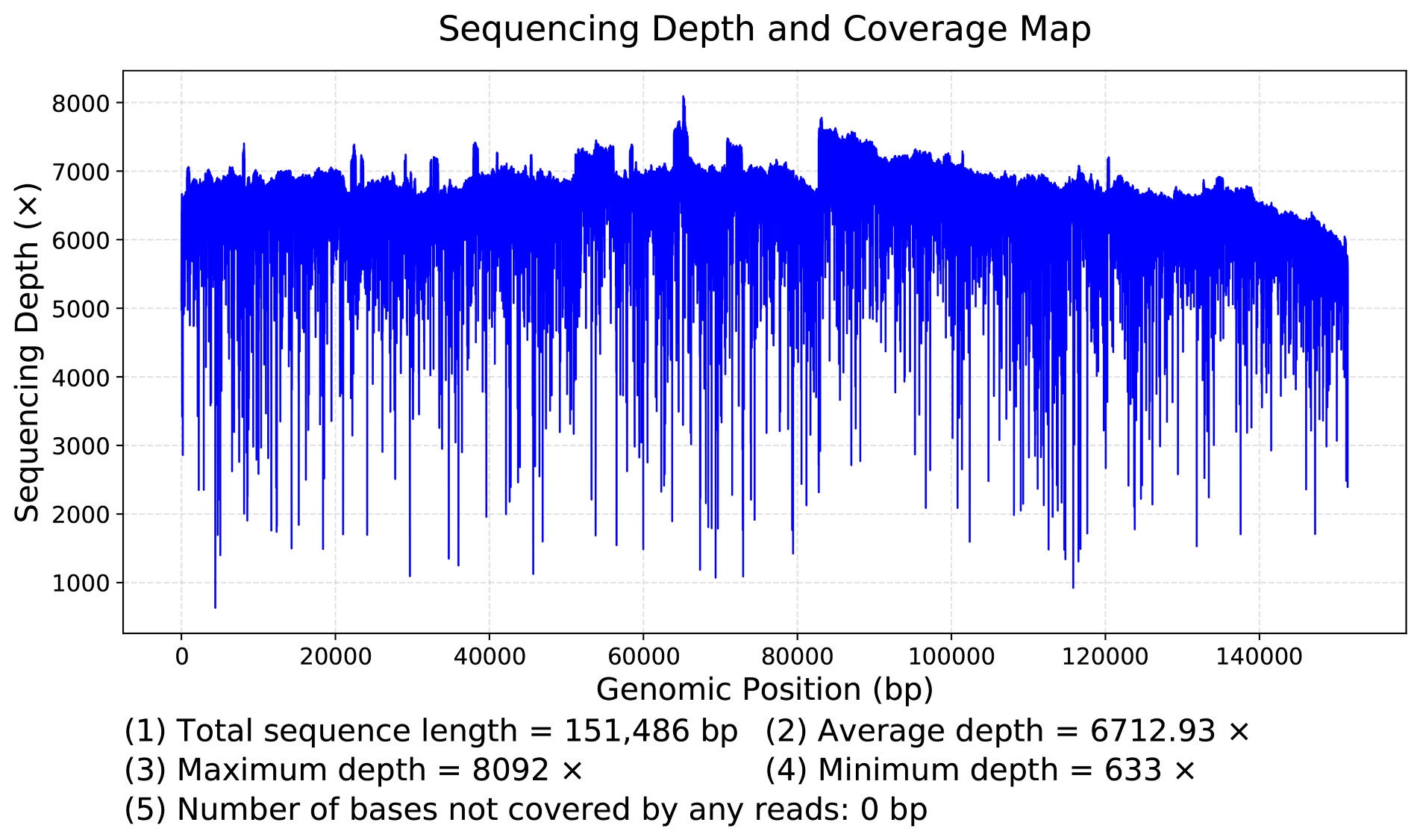


**Figure S2.** Coverage depth of the plastome of *Salvia yunnanensis* based on NGS reads (A) and TGS reads (B). The horizontal coordinate was the positional information of the nucleotide acids and the vertical coordinate was the corresponding coverage depth of each base.


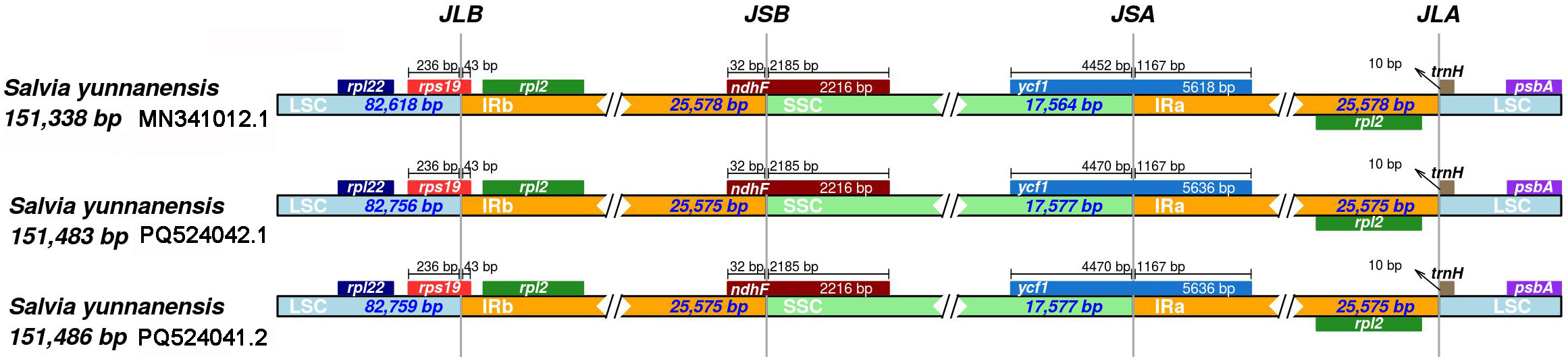


**Figure S3.** Physical mapping of three chloroplast genomes from three different *Salvia* *yunnanensis* individuals.


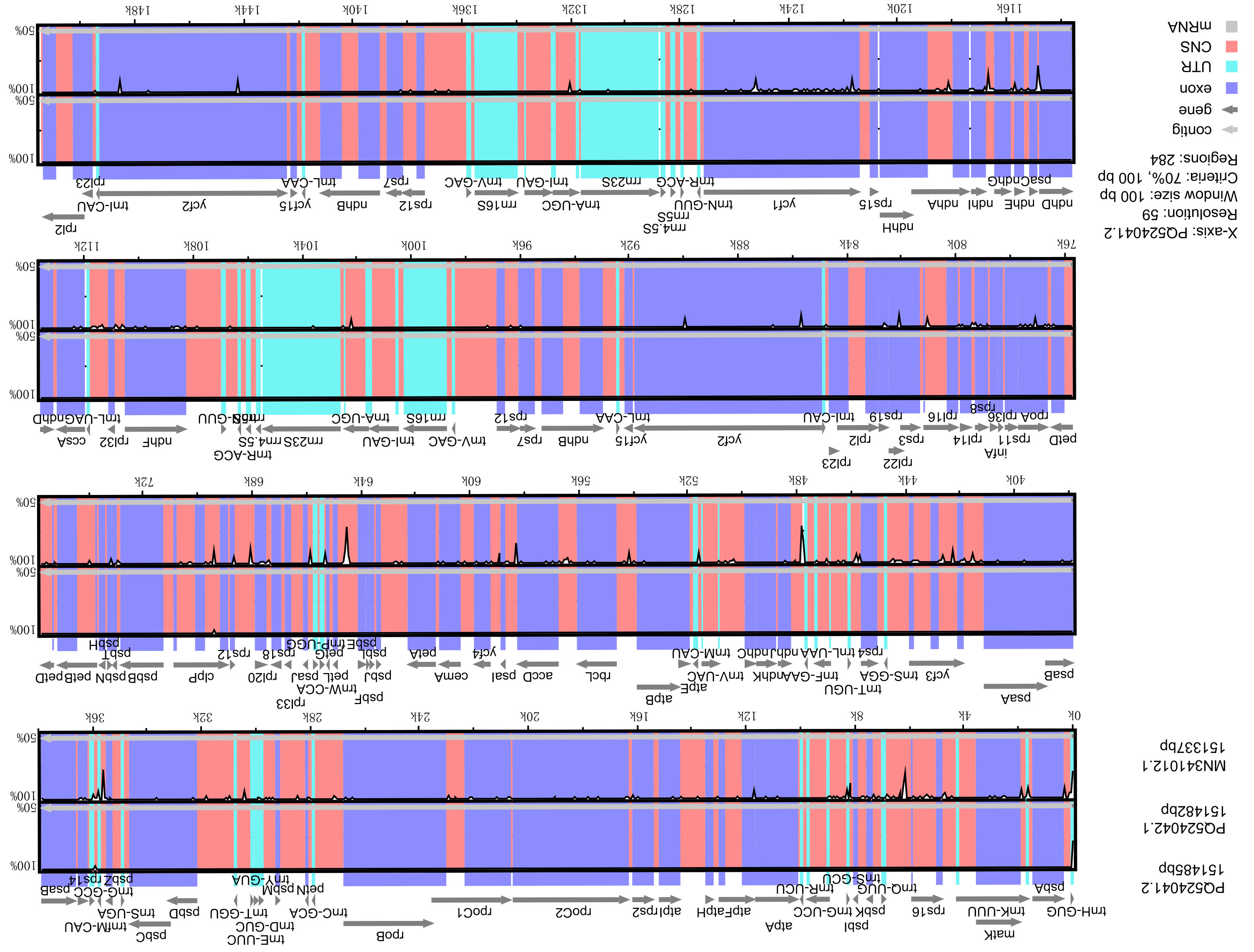


**Figure S4.** mVISTA analysis of three chloroplast genomes from three different *Salvia* *yunnanensis* individuals. Genome labeled by PQ524041.2 was assembled from combined NGS and TGS data in this study. Genome labeled by PQ524042.1 was assembled from NGS data. Genome labeled by MN341012.1 was downloaded from GenBank.


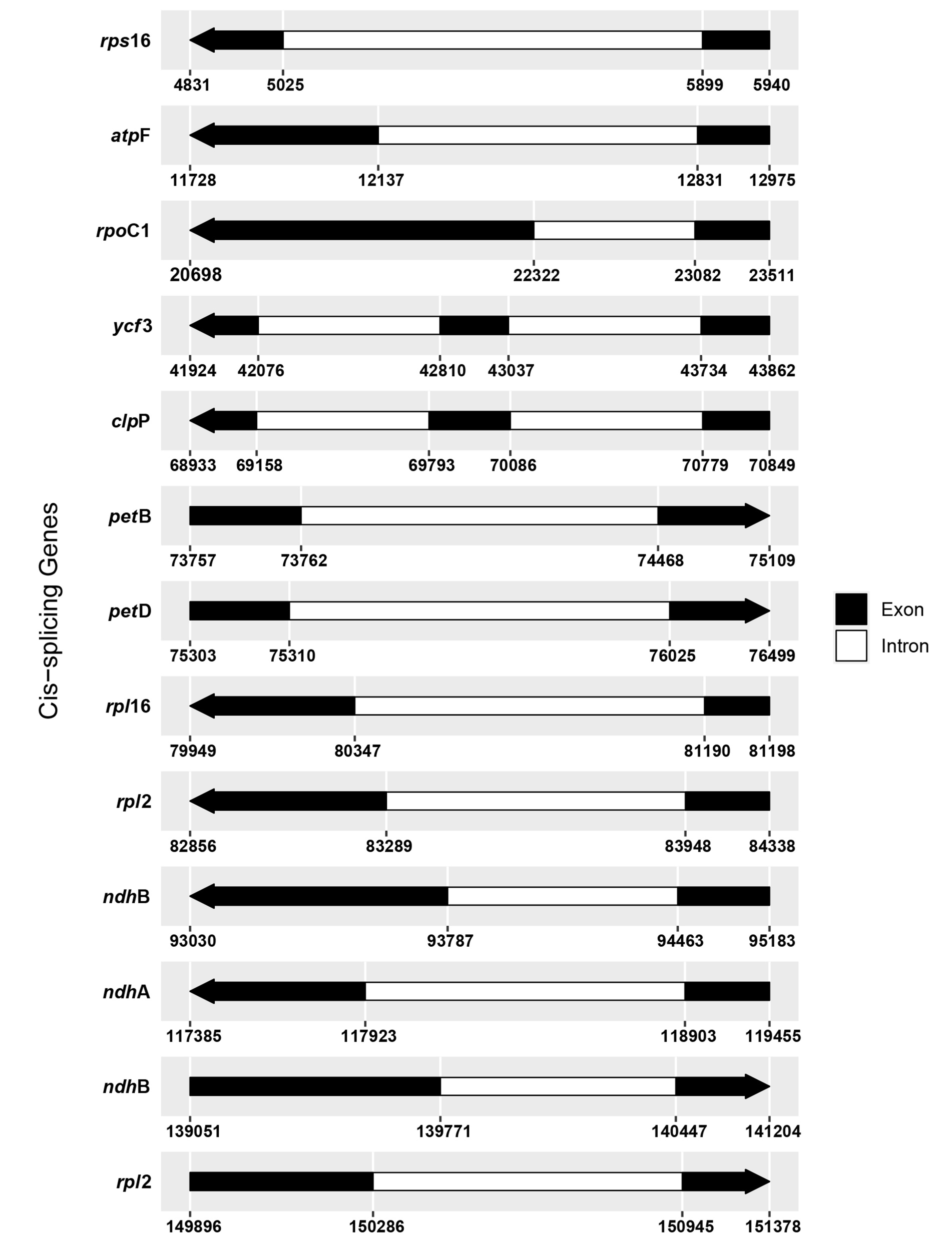


**Figure S5.** Schematic diagram of cis-splicing genes in the plastome of *Salvia yunnanensis*.


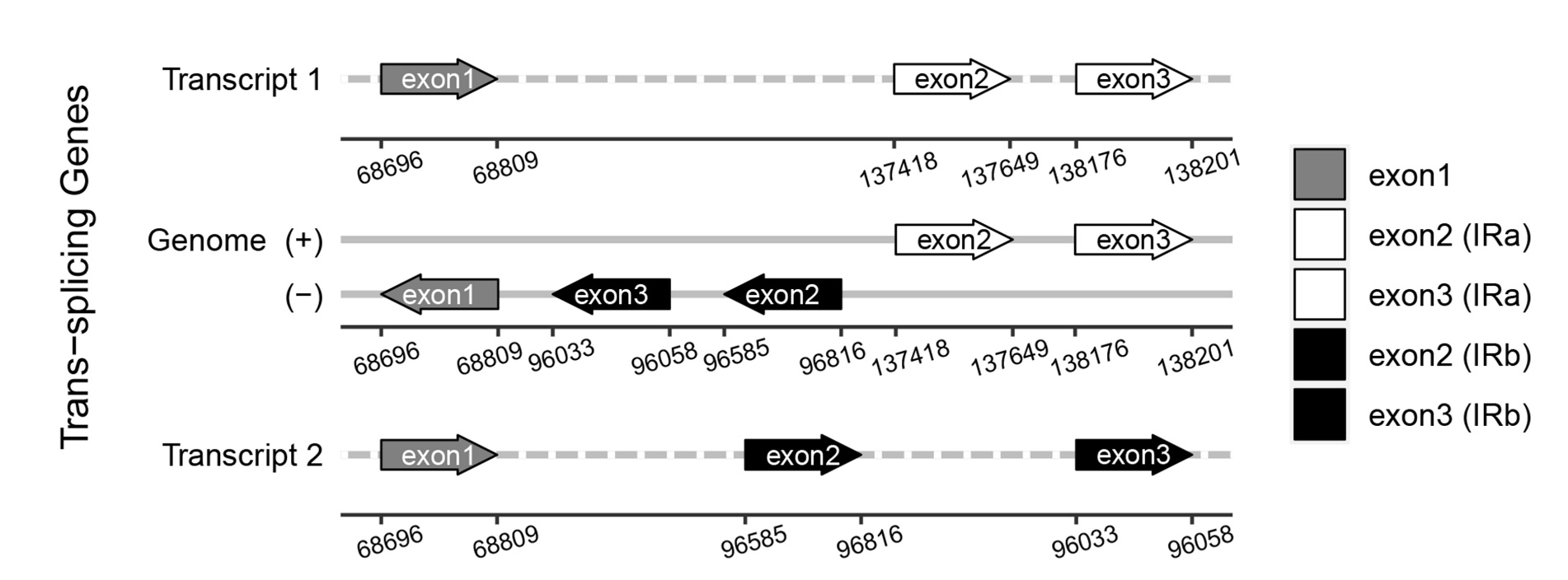


**Figure S6.** Schematic diagram of trans-splicing genes (*rps*12) in the plastome of *Salvia yunnanensis*.





**Figure S7.** The ML phylogenetic phylogram tree of *Salvia yunnanensis* and related taxa based on common gene sequences. The bootstrap values based on 1000 replicates were shown on each node. *Salvia* plants (*n* = 35) and two outgroups were selected for reconstructing the ML tree. New *S. yunnanensis* plastomes (PQ524041, assembled from NGS and TGS data; PQ524042, assembled from NGS data) in this study were labeled in bold font. The two outgroups were *Glechoma longituba* (MK609928.1) (Li and Mo 2019) and *Mentha canadensis* (MN102358.1) (Li *et al*., 2020). The other 33 *Salvia* plastomes were from *S.* *bowleyana* (OM617845.1), *S*. *cavaleriei* var. *simplicifolia* (PP933255.1), *S*. *cavaleriei* (MT634139.1) (Wu H *et al*., 2020), *S*. *prionitis* (MW752214.1, MZ666404.1) (Su *et al*., 2022), *S.* *miltiorrhiza* f. *alba* (MT012420.1), *S.* *miltiorrhiza* (HF586694, JX312195, OR652279) (Qian *et al*., 2013), *S.* *plectranthoides* (MW435409.1, MT634138.1) (Su *et al*., 2022), *S.* *nanchuanensis* (MW435407.1, MZ900990.1), *S.* *nanchuanensis* var. *pteridifolia* (MW435408), *S.* *yunnanensis* (MK944405, MN341012, MN520026, MT634133, MW435411) (Tao *et al*., 2019), *S*. *honania* (MW435406.1, MZ900991.1) (Wang *et al*., 2022), *S.* *plebeia* (MN062352.1, MW381779.1, MW752202.1, MN834131.1) (Cui *et al*., 2020), *S.* *trijuga* (MN062350.1, MN125144.1) (Du *et al*., 2021), *S*. *glutinosa* (MW752204.1) (Huang *et al*., 2024), *S*. *deserta* (MT156378.1, MZ461466.1) (Li *et al*., 2013), *S*. *sclarea* (MN520023.1) (Zhao *et al*., 2020), *S*. *grandifolia* (MT156379.1, MZ461467.1) (Huang *et al*., 2024).


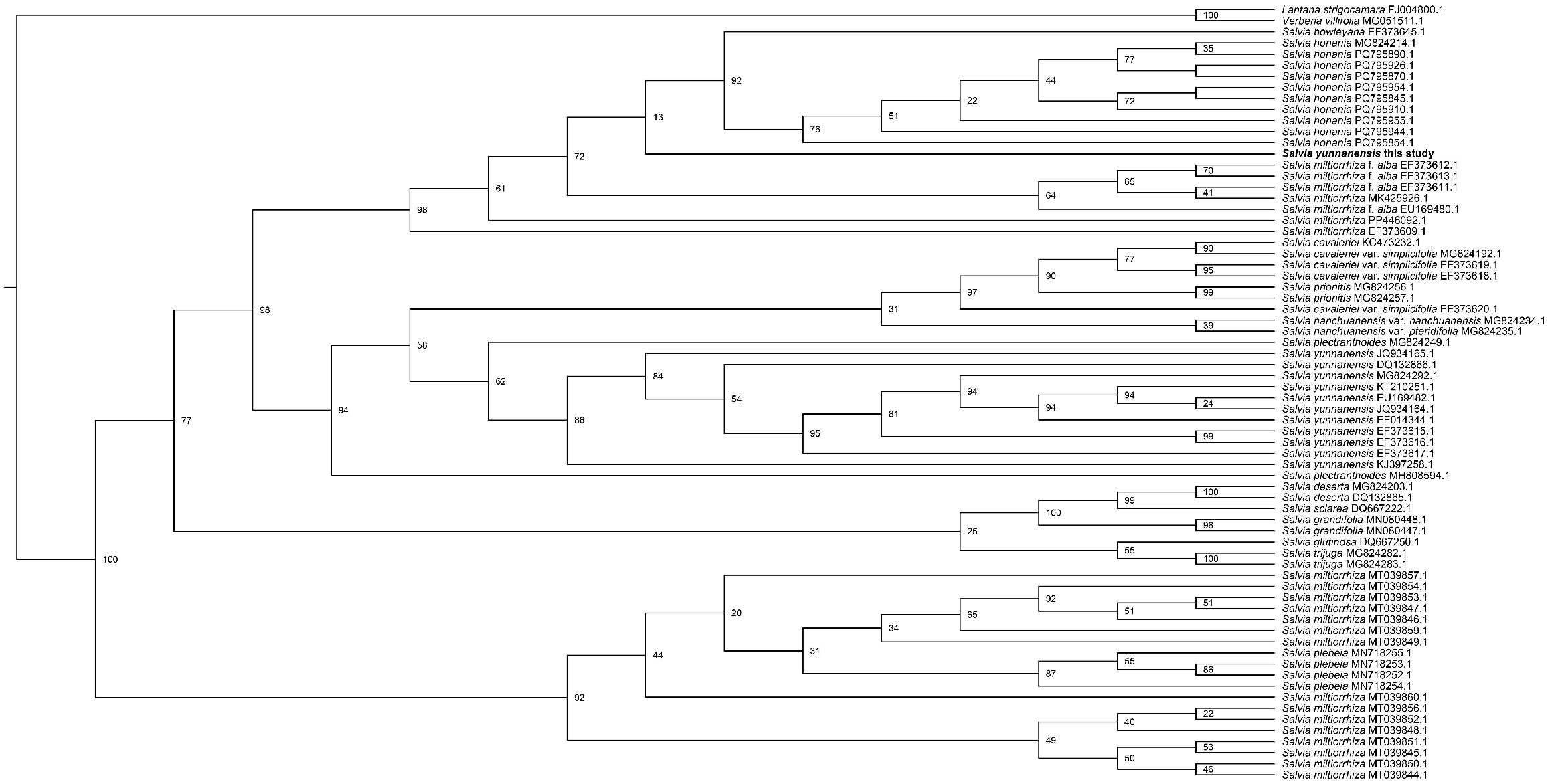


**Figure S8.** The ML phylogenetic phylogram tree of *Salvia yunnanensis* and related taxa based on ITS sequences. The species in this study were highlighted in bold font. The ITS sequences were mainly downloaded from GenBank. The ITS sequence of the *S. yunnanensis* in this study was assembled from the NGS reads (accession number: SRR32111878) using Hybpiper software (https://github.com/mossmatters/HybPiper).
